# A Novel, Rapid, and Low-Volume Assay for Therapeutic Drug Monitoring of Posaconazole and Other Long-Chain Azole-Class Antifungal Drugs

**DOI:** 10.1128/mSphere.00623-18

**Published:** 2018-12-19

**Authors:** Gregory R. Wiedman, Yanan Zhao, David S. Perlin

**Affiliations:** a Public Health Research Institute, New Jersey Medical School-Rutgers Biomedical and Health Sciences, Newark, New Jersey, USA; b Department of Chemistry and Biochemistry, Seton Hall University, South Orange, New Jersey, USA; Carnegie Mellon University

**Keywords:** azole-class antifungal drugs, bioassay, biochemistry, biotechnology, graphene, nanotechnology, synthetic biology, therapeutic drug monitoring

## Abstract

Clinicians need a better way to accurately monitor the concentration of antimicrobials in patient samples. In this report, we describe a novel, low-sample-volume method to monitor the azole-class antifungal drug posaconazole, as well as certain other long-chain azole-class antifungal drugs in human serum samples. Posaconazole represents an important target for therapeutic drug monitoring (TDM) due to its widespread use in treating invasive fungal infections and well-recognized variability of pharmacokinetics. The current “gold standard” requires trough and peak monitoring through high-pressure liquid chromatography (HPLC) or liquid chromatography-tandem mass spectroscopy (LC-MS/MS). Other methods include bioassays that use highly susceptible strains of fungi in culture plates or 96-well formats to monitor concentrations. Currently, no method exists that is both highly accurate in detecting free drug concentrations and is also rapid. Herein, we describe a new method using reduced graphene oxide (rGO) and a fluorescently labeled aptamer, which can accurately assess clinically relevant concentrations of posaconazole and other long-chain azole-class drugs in little more than 1 h in a total volume of 100 µl.

**IMPORTANCE** This work describes an effective assay for TDM of long-chain azole-class antifungal drugs that can be used in diluted human serum samples. This assay will provide a quick, cost-effective method for monitoring concentrations of drugs such as posaconazole that exhibit well-documented pharmacokinetic variability. Our rGO-aptamer assay has the potential to improve health care for those struggling to treat fungal infections in rural or resource-limited setting.

## Abstract (precis)

This work describes an effective assay for TDM of long-chain azole-class antifungal drugs that can be used in diluted human serum samples. This assay will provide a quick, cost-effective method for monitoring concentrations of drugs such as posaconazole that exhibit well-documented pharmacokinetic variability. Our rGO-aptamer assay has the potential to improve health care for those struggling to treat fungal infections in rural or resource-limited setting.

## OBSERVATION

Therapeutic drug monitoring (TDM) of some antimicrobials is critical to optimize pharmacodynamic responses to improve outcomes and reduce the risk for development of drug resistance (1). This is especially important for critically ill and immunosuppressed patients, who are often the target of opportunistic infections (2). Additionally, an important factor to consider is if the drugs being used to treat these patients have a narrow therapeutic window or poorly understood pharmacokinetic variability. The current body of work on monitoring antimicrobials in human serum focuses on two methods: analytical monitoring or bioassays to detect free drug. Most analytical techniques require the separation of the drug from the sample and later detection. These methods effectively define total drug concentration (3). It can be more difficult, however, to tease out the active concentration for highly hydrophobic drugs or those with variable protein binding properties (4, 5). The second method involves the use of highly sensitive drug-susceptible strains of microbes to determine concentration (6). These methodologies, while cost-effective, are limited by the growth rate of the microbes and hence delay concentration determination by up to 48 h in some cases. Fast, accurate, and potentially point-of-care monitoring would be ideal for cases of invasive infections.

In our previous work, we described the development of an aptamer that binds to the azole class of antifungal drugs and its promise as a key component of an assay for antifungals (7). The aptamer we developed, called “R13,” bound to the antifungal drugs posaconazole, fluconazole, voriconazole, and itraconazole. These drugs are lanosterol 1,4-α-demethylase enzyme-inhibiting molecules. Their inhibition of this enzyme prevents the production of ergosterol, which, in turn, weakens the fungal cell wall (8). Some of these drugs, like posaconazole, are difficult to assay in patients due to their high hydrophobicity and various pharmacokinetics. For these reasons, we developed an aptamer that could capture free drug from a sample. Using synthetic evolution of ligands through exponential enrichment (SELEX), we selected an aptamer with high affinity for azoles (9, 10). We also demonstrated that this aptamer could be successfully paired with the nascent technology of graphene field effect transistors (GFETs) to create electrical biosensors (11). We believe that this aptamer could be useful in many other sensing platforms.

We now report on the use of the azole-class antifungal aptamer to detect posaconazole and itraconazole in human serum samples.

### Assay design.

**FIG 1:**
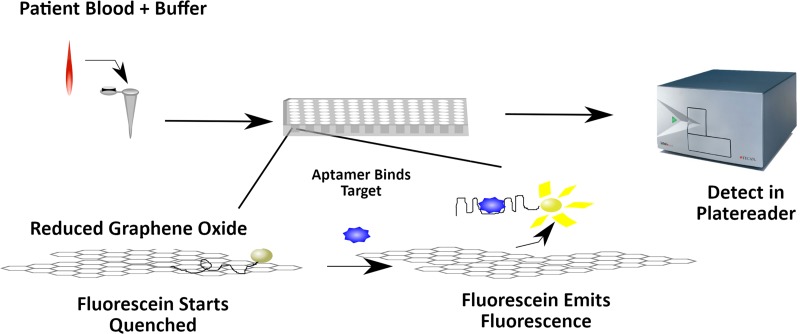
Diagram of the rGO-aptamer assay. The rGO-aptamer assay is implemented by first incubating a fluorescently labeled aptamer with reduced graphene oxide (rGO). A patient sample containing drugs would be mixed with the rGO-aptamer solution in a 96-well plate. This plate would be allowed to incubate for up to an hour and then read in a conventional plate reader.

A detection assay was developed that uses reduced graphene oxide (rGO) and a fluorescently labeled aptamer to detect free drug concentrations (Fig. 1). Fluorescein-labeled aptamers are quenched by rGO in the absence of their target (12). Addition of free target molecules posaconazole and itraconazole liberated the aptamer from the surface, which causes an increase in the fluorescent signal. Serum-bound drug was not detected. This signal was then used to generate concentration curves from human serum samples spiked with drug. Assays were performed in human serum diluted to 50% serum and 10% serum. The concentrations, when adjusted for dilution, were found to be within clinically relevant trough concentrations in clinical studies (*C*_min_) (13). This assay provides a quick and accurate monitoring of free drug concentrations of posaconazole and itraconazole.

### Assay for free posaconazole in buffer and serum.

**FIG 2:**
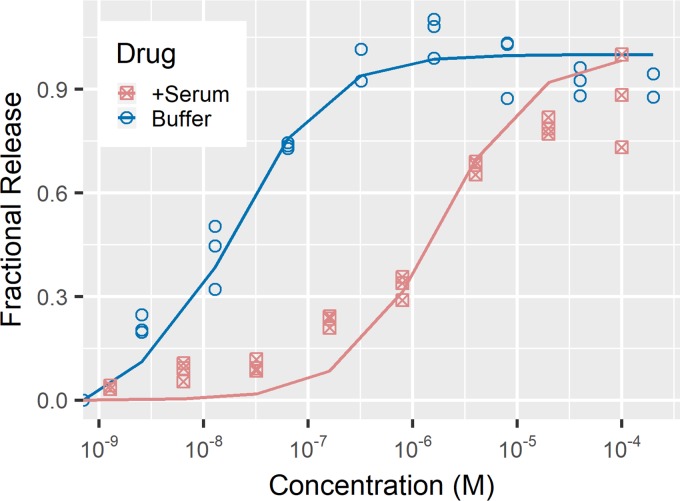
rGO assay in SELEX buffer and 10% human serum. Posaconazole was spiked into SELEX buffer with and without 10% human serum. The change in fluorescein signal was taken to be aptamer released from the surface. Values of fractional release were calculated with respect to the minimum value (with only DMSO) and the maximum value (greatest change in fluorescence). The concentration dynamic range shifts in serum, as expected due to high posaconazole-protein binding.

The rGO-aptamer assay showed a detectable response when tested on samples of posaconazole in SELEX buffer and 10% serum in a 100-µl total volume. Samples spiked with different amounts of posaconazole showed increasing fluorescence intensity with increasing posaconazole concentration (Fig. 2). These data were fit to the first-order reaction equation shown below. The dissociation constant (*K_d_*) was found to be 20 nM. Samples in 50% serum suffered from significant signal-to-noise issues as the minimum detectable concentration was at 5 µM (data not show). The samples tested in 10% serum showed a better response. The dissociation constant for posaconazole in 10% serum was found to be 1.7 µM, almost 100-fold higher than in SELEX buffer. These results are in line with the fact that posaconazole is >99% protein bound in human serum. This suggests that our assay detects free drug in serum. A dynamic range of detection in 10% serum was determined to be 0.224 to 28 µg/ml (0.32 to 40 µM) of posaconazole.

### Selectivity of the posaconazole assay.

**FIG 3:**
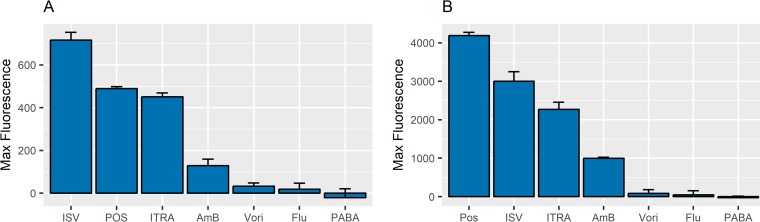
Aptamer released caused by various drug classes. The aptamer release from the rGO surface caused by drugs at 40 µM, as determined by fluorescence values, was measured in SELEX buffer (A) and in SELEX buffer plus 10% serum (B). The azole-class drugs posaconazole (POS), isavuconazole (ISV), and itraconazole (ITRA) liberated the greatest amount of aptamer. Polyene drugs (amphotericin B [AmB]), the azole drugs voriconazole (Vori) and fluconazole (Flu), and the drug *para*-aminobenzoic acid (PABA) elicited small amounts of aptamer release.

The selectivity of the posaconazole assay was examined against the other azole-class antifungal drugs. These drugs were tested at the high concentration of 28 µg/ml (40 µM). Interestingly, in SELEX buffer, only isavuconazole and itraconazole were able to liberate aptamer from the rGO surface and cause an increase in fluorescence (Fig. 3). Two control tests were performed to examine chemical specificity. The highly hydrophobic antifungal drug amphotericin B did not cause a significant signal in this assay in SELEX buffer. It should be noted, however, amphotericin B liberated more aptamer than voriconazole or fluconazole—likely due to the latter’s small size and relative hydrophilic nature. Additionally, a single benzene ring molecule, *para*-aminobenzoic acid (PABA), was unable to cause aptamer release from the surface. Taken together, these data highlight the fact that the assay is not simply influenced by hydrophobicity or a simple structural characteristic (PABA) effect, as previously described (7).

### Assay for itraconazole and isavuconazole in 10% serum.

**FIG 4:**
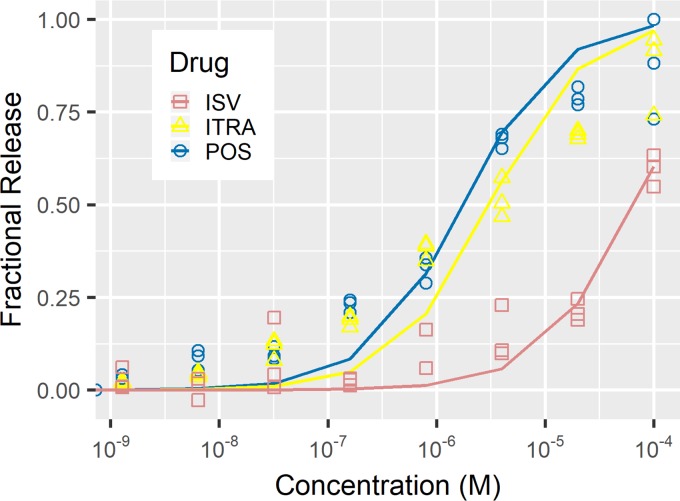
Comparison of aptamer release with azoles. Samples of SELEX buffer plus 10% serum were spiked with three azole-class drugs: posaconazole (POS), itraconazole (ITRA), and isavuconazole (ISV). The assay showed higher sensitivity for posaconazole and itraconazole relative to that for isavuconazole.

We examined the ability of this assay to detect itraconazole and isavuconazole in 10% serum solutions. The apparent detection limit for itraconazole appeared to be the same as that of posaconazole: 0.224 µg/ml (0.32 µM). The apparent detection limit for isavuconazole, however, appeared to be greater at above 1.12 µg/ml (1.6 µM). Fitting to a first-order reaction equation was done by taking a control well of 100 µM posaconazole to be the maximum release. The dissociation constants for itraconazole and isavuconazole were found to be 3 µM and 65 µM, respectively, in 10% serum (Fig. 4). The value for itraconazole was in the range of that found for posaconazole, but isavuconazole was almost 40 times higher. Taken together, these data suggest that the best utility of this assay is in detecting posaconazole and itraconazole from diluted serum solutions.

Pharmacokinetic variability is well established from certain azole-class antifungal agents like posaconazole (14), and successful patient management requires careful TDM. Conventional analytical approaches are cumbersome, slow, and require high-end instrumentation. Better methods are needed for TDM of drugs like posaconazole, isavuconazole, and itraconazole. These methods should have the potential to be used at bedside and in resource-limiting settings. Our assay has expanded the repertoire of existing methods by adding a way to quickly detect the concentration of posaconazole and other long-chain azole-class antifungal drugs in 10% serum solutions in 1 h or less from several drops of liquid. When adjusted for dilution, the effective drug measurement is within an almost 100-fold range from 0.224 to ∼28 µg/ml (0.32 to 40 µM). Previous reports have noted that trough concentrations for treating azole-susceptible Aspergillus infections are around 0.2 µg/ml and that therapeutic concentrations for other invasive fungal infections can be upwards of 1.5 µg/ml (13, 15). Peak concentrations may even reach the higher end of this range, making this assay extremely useful to clinicians (16). The assay is highly specific as only posaconazole and itraconazole caused significant signals, along with isavuconazole to a lesser extent. This observation is consistent with the structural overlap between posaconazole and itraconazole (17).

In summary, the reduced graphene oxide-aptamer assay provides a promising new analytical detection platform for therapeutic drug monitoring of certain azole-class antifungal drugs.

### Materials.

Human serum and posaconazole were purchased from Sigma-Aldrich (St. Louis, MO). Fluconazole and other azole drugs were purchased from Santa Cruz Biotechnologies (Dallas, TX). Reduced graphene oxide was purchased from Graphenea (San Sebastián, Spain). Fluorescein-labeled aptamers were synthesized by LGC Biosearch Technologies (Petaluma, CA). All other reagents and solvents were purchased from Thermo Fisher Scientific (Waltham, MA).

### Preparing rGO-aptamer mixtures.

The rGO-Apatamer mixtures were made by first solubilizing 30 nmol of fluorescein-labeled aptamer in 1× SELEX buffer (14 mM sodium chloride, 0.2 mM potassium chloride, 0.5 mM magnesium chloride, 0.2 mM calcium chloride in 2 mM pH 7.4 Tris). The aptamer DNA was then heated at 95°C for 5 min, placed on ice for 15 min, and then incubated at room temperature (25°C) for 5 min. A stock solution of 1 mg/ml rGO was prepared in either 1× SELEX buffer or 1× SELEX buffer with 10% human serum. The DNA was incubated with 150 µl of the 1 mg/ml rGO for 15 min at room temperature with shaking. The sample was then spun down at 5,000 × *g* for 1 min. The supernatant was removed, and the graphene pellet was washed once with either 1× SELEX buffer or 1× SELEX buffer with 10% human serum.

### Fluorescence assay.

The fluorescence assays in this work were conducted in 96-well Sarstedt plates (Nümbrecht, Germany). Dilutions of azole drugs were prepared in dimethyl sulfoxide (DMSO) and pipetted individually into wells of the plates. The amount of DMSO was a constant 1 µl. After this, 50 µl of buffer (SELEX or SELEX with 10% serum) was added to each well. Samples of rGO that had been incubated with aptamer and washed were solubilized into 1.5 ml of the appropriate buffer. Then 30 wells of a 96-well plate received 50-μl aliquots of this sample. Ultimately, each well contained 5 μl of 1 mg/ml of graphene.

Samples were then allowed to incubate for up to 1 h. After 1 h, the plates were placed into a Tecan infinite M200Pro plate reader. The samples were read in fluorescence mode with excitation at 494 nm and emission at 525 nm. Sample fluorescence values were analyzed in Microsoft Excel and R 3.5.0. These values were normalized to a background well containing only 1 µl of DMSO and a maximum control well containing 100 µM posaconazole. Data curves were fit to a standard first-order reaction equation, *F_r_*(*C*) = *C*/(*C* + *K_d_*), where the fraction released (*F_r_*) with respect to concentration (*C*) is a function of the concentration and the dissociation constant (*K_d_*).
